# Roles of Response Regulators in the Two-Component System in the Formation of Stress Tolerance, Motility and Biofilm in *Salmonella* Enteritidis

**DOI:** 10.3390/foods13223709

**Published:** 2024-11-20

**Authors:** Mengjun Hu, Zhuoan Zhou, Chenqi Liu, Zeqiang Zhan, Yan Cui, Shoukui He, Xianming Shi

**Affiliations:** MOST-USDA Joint Research Center for Food Safety, State Key Laboratory of Microbial Metabolism, School of Agriculture and Biology, Shanghai Jiao Tong University, Shanghai 200240, China; lvysjtu2018@sjtu.edu.cn (M.H.); zza19991218@163.com (Z.Z.); 19909lcq@sjtu.edu.cn (C.L.); zhanzeqiang@sjtu.edu.cn (Z.Z.); cyan9028@163.com (Y.C.); heshoukui@sjtu.edu.cn (S.H.)

**Keywords:** *S*. Enteritidis, TCS, response regulator, environmental stress, biofilm matrix

## Abstract

Two-component systems (TCS) of *Salmonella enterica* serovar Enteritidis are composed of a histidine kinase and a response regulator (RR) and represent a critical mechanism by which bacteria develop resistance to environmental stress. Here, we characterized the functions of RRs in TCS in the formation of stress tolerance, motility and biofilm using twenty-six *S*. Enteritidis RR-encoding gene deletion mutants. The viability results unraveled their essential roles in resistance to elevated temperature (GlrR), pH alterations (GlrR, TctD, YedW, ArcA and YehT), high salt (PhoB, BaeR, CpxR, PhoP, UvrY and TctD), oxidative stress (PhoB, YedW, BaeR, ArcA, PhoP, UvrY, PgtA and QseB) and motility (ArcA, GlnG, PgtA, PhoB, UhpA, OmpR, UvrY and QseB) of *S*. Enteritidis. The results of the crystal violet staining, microscopy observation and Congo red binding assays demonstrated that the absence of ArcA, GlnG, PhoP, OmpR, ZraR or SsrB in *S*. Enteritidis led to a reduction in biofilms and an impairment in red/dry/rough macrocolony formation, whereas the absence of UvrY exhibited an increase in biofilms and formed a brown/smooth/sticky macrocolony. The results indicated the regulatory effects of these RRs on the production of biofilm matrix, curli fimbriae and cellulose. Our findings yielded insights into the role of TCSs, making them a promising target for combating *S*. Enteritidis.

## 1. Introduction

*Salmonella* is a common Gram-negative foodborne bacterial pathogen responsible for the highest number of outbreaks and cases [1]. An evaluation of foodborne salmonellosis contamination routes by the World Health Organization (WHO) suggests that *Salmonella* infections caused by contaminated eggs and poultry are prevalent in 14 regions worldwide [2]. There are more than 2600 *Salmonella* serotypes identified, of which *Salmonella* Enteritidis (*S*. Enteritidis) is the most frequent one associated with the reported foodborne outbreaks (67.3%) [1,3]. Infection with *S*. Enteritidis often manifests as abdominal pain, diarrhea and self-limiting gastroenteritis; it even causes systemic infections such as sepsis [4], posing a threat to food safety.

*S*. Enteritidis commonly encounters a range of adverse environmental stresses during industrial production, including extremes of temperature, pH, osmotic pressure and the presence of reactive oxygen species (ROSs), which have an adverse effect on the physiological welfare of bacterial cells, leading to inhibition and/or death at a cellular or a populational level [5]. Currently, many food preservation techniques operate on this idea by introducing one or more stresses that prevent bacterial development in food. However, *S*. Enteritidis has adapted to survive in a wide range of different environments via metabolic and/or genotypic alterations, such as the changes in the cell wall/membranes and their proteins (e.g., porins and efflux pumps) [5,6,7,8]. In addition, biofilm formation and motility are required for colonization and are associated with the persistence of *S*. Enteritidis in food supply chains [9,10]. It has been demonstrated that biofilm-encased *Salmonella* exhibits resistance to eradication by chemical, physical and mechanical stresses [9]. Therefore, more in-depth studies are needed to investigate the underlying molecular mechanism of *S*. Enteritidis stress tolerance on aspects of microbial physiology.

In prokaryotic organisms, signal transduction systems play a key role in the regulatory circuits that modulate bacterial physiology in response to changing conditions. Two-component systems (TCSs) have been subjected to intensive research since their discovery [11]. A canonical TCS consists of an inner membrane-embedded histidine sensor kinase (HK) and a cytosolic response regulator (RR). When HK is activated by environmental stress, it auto-phosphorylates and then transfers phosphoryl groups to its cognate RR [12,13]. The activated RR binds specifically to the promoters of target genes to regulate their transcription, thereby initiating cellular responses and defending against adverse environment stresses [12,13]. Accordingly, deletion of a RR-encoding gene would directly abolish the function of a TCS. TCSs are involved in most aspects of bacterial physiology, including stress response, motility, biofilm, antibiotic resistance, nutrient uptake, central metabolism and virulence [14,15,16]. For instance, in *Pseudomonas aeruginosa* (*P. aeruginosa*), there are fifty-six TCSs involved in fimbriae synthesis that contribute to biofilm formation [17]; in *Salmonella*, PmrB-PmrA and PhoQ-PhoP are the core TCSs involved in regulating the expression of lipid A modification genes and operons (e.g., *arnBCADTEF*-*ugd* and *eptB*), whereby changing the cell surface chargeability and hydrophobicity, and increasing *Salmonella* resistance to polymyxins [18]. Although certain TCSs have been studied and characterized, the regulatory details of homologous TCSs differ between species. TCS EnvZ-OmpR, for example, directly controls the expression of the flagellar and curli production-related operons *flhDC* and *csgDEFG* in *Escherichia coli* (*E. coli*), the cryptic porin genes *ompS1* and *ompS2* in *Salmonella* Typhi (*S.* Typhi), and the tetrathionate-metabolic gene *tetH* in *Acidithiobacillus*, which contributes to different phenotypes, including biofilm formation, pathogenicity and high-salt stress adaptation, respectively [19,20,21]. However, the roles of TCSs in stress response and their regulation have not been investigated in *S.* Enteritidis.

Thirty TCSs in *S.* Enteritidis were identified by genomic sequencing in our previous study [22]. Additionally, antibiotic susceptibility tests on the generated twenty-six *S*. Enteritidis RR-encoding gene deletion mutants (RR mutants in briefly) showed that ten RRs in TCS had a role in resistance to antibiotics [22]. However, little is known about their roles in other physiological characteristics. In this study, the twenty-six RR mutants were subjected to an evaluation process to ascertain their adaptability to a diverse range of environmental stresses, as well as for their motility and biofilm formation capacity. This study provided valuable information details of certain *S.* Enteritidis TCSs and highlighted their role in modulating the stress tolerance response, motility and biofilm formation, which will aid future research into the signals and underlying mechanisms involved in *S.* Enteritidis tolerance processes.

## 2. Materials and Methods

### 2.1. Bacterial Strains and Culture Conditions

*S*. Enteritidis SJTUF12367 (GenBank accession no. GCA_004323915.1; https://www.ncbi.nlm.nih.gov/assembly/GCF_004323915.1/, accessed on 4 March 2019) and its derivatives (twenty-six RR mutants) [22] were used in this study. *Salmonella* Enteritidis SJTUF12367 was provided by the Shanghai Municipal Center for Disease Control and Prevention and isolated from a fecal sample of a patient who had consumed food contaminated with the bacterium. All the strains were stored in lysogeny broth (LB) medium (Land Bridge, Beijing, China) with 50% (*v*/*v*) glycerol (Aladdin, Shanghai, China) at −80 °C. These cells were streaked on LB agar plates (Land Bridge, Beijing, China) and cultured at 37 °C. Individual colonies were incubated in 5 mL of LB at 37 °C with 180 rpm shaking. For subsequent use, the suspensions of overnight cultured bacteria were diluted with fresh LB broth with an adjusted density of OD_600_ of 1.0.

### 2.2. High Temperature and Osmotically Stressed Assays

The high temperature and osmotically stressed assays were performed as previously described with modifications [6,21]. For the high temperature (HT) assay, the bacterial suspensions (OD_600_ = 1.0) were inoculated at a dilution of 1:100 into 5 mL of LB and treated at a temperature of 50 °C for 1.5 h in a thermostatic water bath. For the high osmotically (HO) stressed assay, the bacterial suspensions were inoculated at a dilution of 1:100 into 5 mL of LB containing 6% NaCl (Sinopharm, Shanghai, China) at 37 °C under shaking aerobic conditions (180 rpm, for 1 h). The treated bacterial cells were then serial decimal diluted with PBS (pH 7.2) (Beyotime, Shanghai, China) and plated on LB agar plates to calculate the viable cells. Both assays were performed with three biological replicates. The number of colonies obtained in high temperature/osmotic stress at 0 h was used to normalize the results.

### 2.3. Acid and Alkaline Stress Assays

According to He et al. [6], the bacterial suspensions (OD_600_ = 1.0) were inoculated at a dilution of 1:100 into 5 mL of LB with acid (final pH = 4.4) or LB with alkaline (final pH = 9.0), respectively. Following an incubation period at 37 °C under shaking aerobic conditions (180 rpm, for 1 h), the treated bacterial cells were evenly spread on LB agar plates to count the viable cells. Both assays were performed with three biological replicates. The number of colonies obtained in acid/alkaline stress at 0 h was used to normalize the results.

### 2.4. Oxidative Stress Assay

The oxidative stress assay was performed as previously described with modifications [23]. In brief, the bacterial suspensions (OD_600_ = 1.0) were inoculated at a dilution of 1:100 into 5 mL of LB containing 5 mM hydrogen peroxide (H_2_O_2_) (Sinopharm, Shanghai, China). Following an incubation period at 37 °C under shaking aerobic conditions (180 rpm, for 1 h), the treated bacterial cells were spread on LB agar plates to count the viable cells. The assay was performed with three biological replicates. The number of colonies obtained in oxidative stress at 0 h was used to normalize the results.

### 2.5. Motility Assay

The swimming and swarming motility assays were performed as previously described with modifications [24]. The overnight bacterial cultures were diluted at 1:1000 in fresh LB broth, then inoculated with a volume of 0.25 µL or dripped with a volume of 2.5 µL in the center of the LB plate containing 0.3% or 0.6% agar, respectively, which was followed by an incubation at 37 °C for 8 or 16 h. The motility diameters were measured. The assay was performed with six technical replicates.

### 2.6. Determination of Biofilm Formation Ability

The overnight bacteria cultures were diluted at 1:1000 into fresh salt-free LB broth. The tissue culture 96-well polystyrene microplate with 200 µL/well of diluted bacteria cultures was placed in a 28 °C-cultivating box for 48 h, and then the medium was removed [25]. The wells were washed three times with 200 µL of sterile water, and adherent cells were fixed with anhydrous methanol (Sinopharm, Shanghai, China) for 15 min and stained with 200 µL of 0.1% crystal violet (Sinopharm, Shanghai, China). The unattached dye was then gently removed by washing the plate three times with sterile water. After air-drying the plate, 200 µL of 33% acetic acid (Sinopharm, Shanghai, China) was added to each well to dissolve the crystal violet, and biofilm production was estimated by measuring OD_595_.

### 2.7. Microscopic Examination

To observe the biofilm characteristics, the glass coverslips, with a dimension of 2 cm^2^, were sterilized and placed in the center of the 6-well plate with 6 mL of diluted bacteria cultures. The plates were placed in a 28 °C-cultivating box for 48 h, and then the biofilm would adhere to the coverslip’s surface. The medium was removed, and each well was washed three times with 6 mL of sterile water. The coverslips were stained with 6 mL of 0.1% crystal violet. The unattached dye was then gently removed by washing the plate three times with sterile water. After that, they were dried and visualized using an optical microscope (Olympus, Tokyo, Japan) and scanning electron microscope (SEM) (FEI, OR, USA).

### 2.8. Congo Red and Coomassie Brilliant Blue Binding Assays

Congo red (CR) and Coomassie brilliant blue (CBB)-supplemented agars (Aladdin, Shanghai, China) were used to visualize curli and cellulose production in the RR deletion mutants [26]. The overnight cultures (10 µL) were dropped onto salt-free LB agar containing 40 µg/mL of CR or 20 µg/mL of CBB and cultured for 48 h at 28 °C. CR can label large complexes like cellulose or amyloid structures, while CBB stains proteins in the colony biofilms. On the CR agar surface, red, dry and rough colonies were observed for the strains producing curli and cellulose; smooth, pink colonies were observed for strains producing cellulose but no curli; smooth, white colonies were observed for strains producing no cellulose and curli; and stick colonies were observed for strains producing extracellular matrix.

### 2.9. Statistical Analysis

GraphPad Prism 8.0.1 (244) (GraphPad Software, CA, USA) was used to create the graphs, and one-way ANOVA was used for statistical difference analysis; *p*-values of <0.05 were considered statistically significant.

## 3. Results

### 3.1. Role of RRs in TCS in High Temperatures and Osmotic Tolerance of S. *Enteritidis*

**Figure 1:**
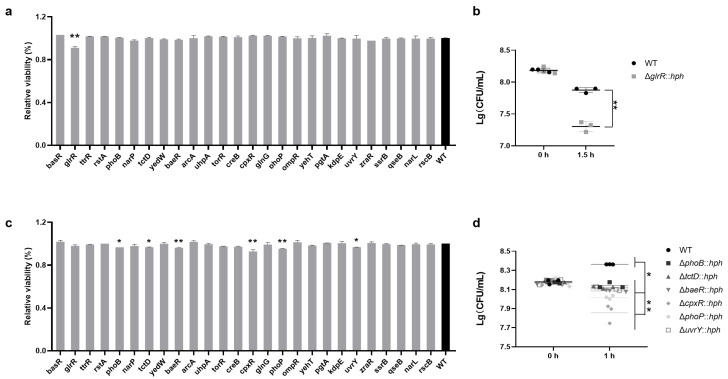
The viability of *S*. Enteritidis WT and its RR mutants in high temperature and high osmolarity stresses. (**a**) The relative viability of *S*. Enteritidis strains (relative to WT) that survived at 50 °C and (**c**) 6% NaCl stresses. The various RR mutants were labeled according to the RR-encoding gene on the *X*-axis. Data were presented as mean ± standard deviation; (**b**) Enumeration of the CFUs of *S*. Enteritidis strains that survived at 50 °C and (**d**) 6% NaCl stresses. Horizontal lines marked the median value for each strain group. The abbreviation ‘*hph*’ was employed as a screening marker for hygromycin resistance in mutants. ‘*’, *p* < 0.05; ‘**’, *p* < 0.01.

*S*. Enteritidis often encounters water activity (Aw) changes in the food matrix and/or the elevation and depression of temperature as food preservation progresses. In this study, the viability of *S*. Enteritidis strains was similar when cultured at 37 °C for 1.5 h, indicating that these RRs were not essential for *S*. Enteritidis’ short-term growth in normal temperature conditions (Figure S1). However, compared to the WT, the absence of GlrR manifested a growth defect at 50 °C, indicating that GlrR played a crucial role in the HT tolerance of *S*. Enteritidis (Figure 1a,c). Meanwhile, compared to the WT, the absence of PhoB, TctD, BaeR, CpxR, PhoP or UvrY led to a significant (*p* < 0.05 or *p* < 0.01) decrease in viability under HO pressure (Figure 1b,d), suggesting a positive role of these RRs in defending the osmotic stress of *S*. Enteritidis.

### 3.2. Role of RRs in TCS in Acid and Alkali Tolerance of S. *Enteritidis*

**Figure 2:**
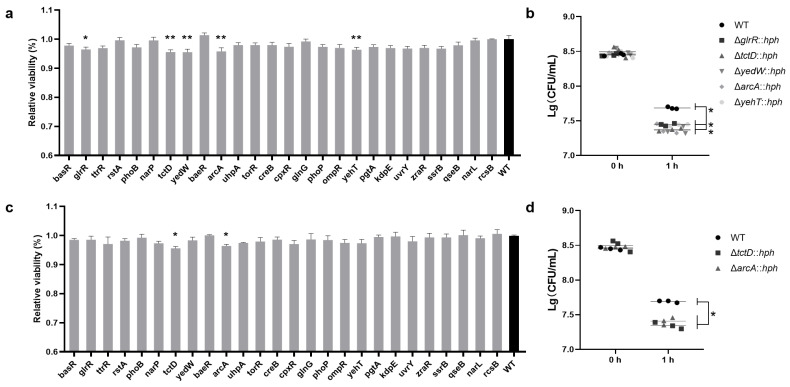
The viability of *S*. Enteritidis WT and its RR mutants in acid and alkali stresses. (**a**) The relative viability of *S*. Enteritidis strains (relative to WT) that survived at pH 4.0 and (**c**) pH 9.0 stresses. The various RR mutants were labeled according to the RR-encoding gene on the *X*-axis. Data were presented as mean ± standard deviation; (**b**) Enumeration of the CFUs of *S*. Enteritidis strains that survived at pH 4.0 and (**d**) pH 9.0 stresses. Horizontal lines marked the median value for each strain group. The abbreviation ‘*hph*’ was employed as a screening marker for hygromycin resistance in mutants. ‘*’, *p* < 0.05; ‘**’, *p* < 0.01.

To examine the functions of RRs in TCS in the formation of acid and alkali tolerance, the *S*. Enteritidis WT and twenty-six RR mutants were cultured in LB broth at a pH 4.4 or 9.0. All the mutants were inoculated with an initial amount equivalent to that of the *S*. Enteritidis WT. A comparison of the initial colony-forming units (CFU) value at 0 h with the CFU value of either *S*. Enteritidis WT or mutants grown at pH 4.4 or 9.0 for 1 h revealed a decrease in the latter (Figure 2). Compared to the WT, five RR (ArcA, TctD, YedW, GlrR and YehT) mutants demonstrated a significantly diminished viability (*p* < 0.01) at pH 4.4 (Figure 2a,b). It was notable that the absence of ArcA or TctD resulted in a more pronounced defect in viability than that observed with the other three RRs. Furthermore, the absence of ArcA or TctD also led to a significant (*p* < 0.01) decrease in viability when strains were grown at pH 9.0 (Figure 2c,d). Therefore, it can be concluded that the RRs of ArcA and TctD played a prominent role in the adaptation of *S*. Enteritidis to pH changes.

### 3.3. Roles of RRs in TCS in Oxidative Tolerance of S. *Enteritidis*

**Figure 3:**
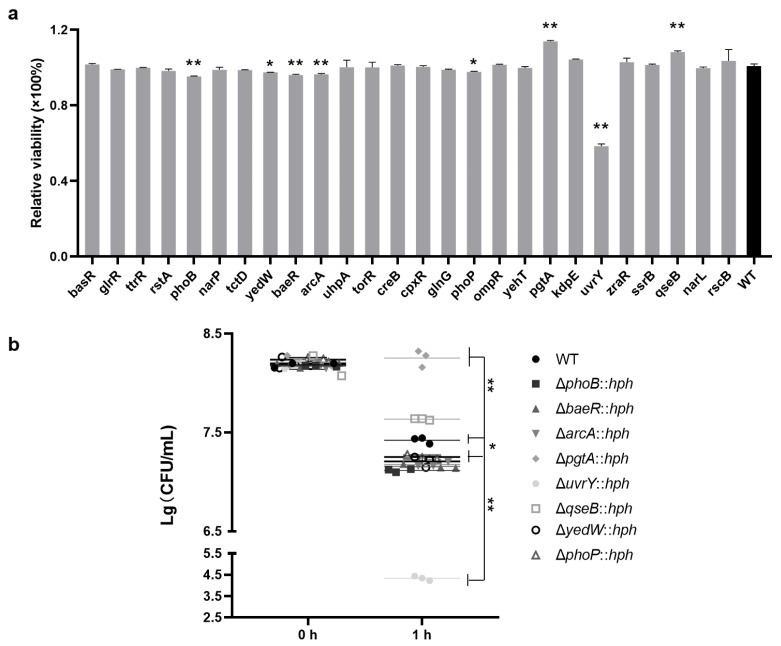
The viability of *S*. Enteritidis WT and its RR mutants in oxidation stress. (**a**) The relative viability of *S*. Enteritidis strains (relative to WT) that survived at 5 mM H_2_O_2_ stress. The various RR mutants were labeled according to the RR-encoding gene on the *X*-axis. Data were presented as mean ± standard deviation; (**b**) Enumeration of the CFUs of *S*. Enteritidis strains that survived at 5 mM H_2_O_2_ stress. Horizontal lines marked the median value for each strain group. The abbreviation ‘*hph*’ was employed as a screening marker for hygromycin resistance in mutants. ‘*’, *p* < 0.05; ‘**’, *p* < 0.01.

The cell viability of *S*. Enteritidis WT and the RR mutants grown in the presence of H_2_O_2_ were also determined. The viability of six RR (PhoB, YedW, BaeR, ArcA, PhoP and UvrY) mutants were significantly (*p* < 0.05 or *p* < 0.01) decreased in the presence of H_2_O_2_ (Figure 3a), indicating a positive regulation in oxidative tolerance. In contrast, the absence of PgtA or QseB resulted in a significant (*p* < 0.01) increase in viability than that of WT, suggesting a negative regulation. Strikingly, the absence of UvrY had the greatest impact on *S*. Enteritidis, resulting in a 3.09-log reduction in viable cell counts following exposure to H_2_O_2_ (Figure 3b). This observation suggested that UvrY played a pivotal role in oxidative stress tolerance.

### 3.4. Roles of RRs in TCS in Motility of S. *Enteritidis*

**Figure 4:**
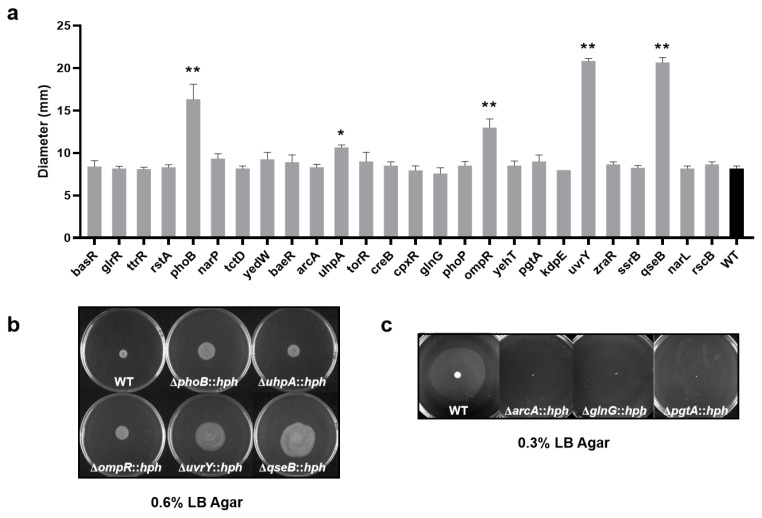
The motility of *S*. Enteritidis WT and its RR mutants. (**a**) The diameters of the swarming zone of *S*. Enteritidis strains on 0.6% agar plates. The various RR mutants were labeled according to the RR-encoding gene on the *X*-axis. Data were presented as mean ± standard deviation; (**b**) The swarm agar plates of *S*. Enteritidis WT and five RR mutants; (**c**) The swim agar plates of *S*. Enteritidis WT and three RR mutants. The abbreviation ‘*hph*’ was employed as a screening marker for hygromycin resistance in mutants. The swarming and swimming morphology images were taken after 16 h or 8 h, respectively. ‘*’, *p* < 0.05; ‘**’, *p* < 0.01.

Most of the RR mutants exhibited similar motility on swarm (21/26) or swim (23/26) agar plates, respectively. However, several RRs have the capacity to alter the motility via transcriptional regulation in *S*. Enteritidis. As shown in Figure 4a,b, the *S*. Enteritidis WT strain could swarm via the combined effects of flagellar motility, chemotaxis and growth, thus creating a circular colony on a 0.6% agar plate (approximately 8.5 mm in diameter), whereas five RR (PhoB, UhpA, OmpR, UvrY and QseB) mutants were dispersed significantly (*p* < 0.05) wider than that of WT (approximately 10.5~21.0 mm in diameter), indicating roles of these RRs in repressing cell motility. In addition, three RR (ArcA, GlnG and PgtA) mutants exhibited notably inhibited swimming motility with no dispersal (Figure 4c), indicating their roles in promoting cell motility.

### 3.5. Roles of RRs in TCS in Biofilm Formation of S. *Enteritidis*

**Figure 5:**
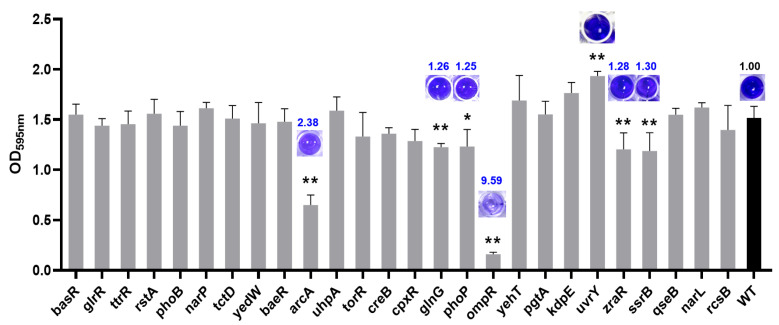
The biofilm formation capacity of *S*. Enteritidis WT and its RR mutants. The various RR mutants were labeled according to the RR-encoding gene on the *X*-axis. Data were presented as the average of five samples, and error bars represent the standard deviation. ‘*’, *p* < 0.05; ‘**’, *p* < 0.01.

To determine whether RRs in TCS influence the formation of *Salmonella* biofilms, a crystal violet staining procedure was performed to ascertain the ability of *S*. Enteritidis to attach to plastic surfaces (Figure 5). The results showed that compared to the WT, the absence of RR UvrY resulted in an increase in biofilm formation (*p* < 0.01), while the absence of RRs of ArcA, GlnG, PhoP, OmpR, ZraR or SsrB led to significantly decreased biofilm production (*p* < 0.01 or *p* < 0.05). Of note, the lack of ArcA or OmpR resulted in a significant reduction in biofilm formation, with a 2.38- or 9.59-fold decrease, respectively. This suggested that these two RRs played a pivotal role in regulating biofilm formation.

**Figure 6:**
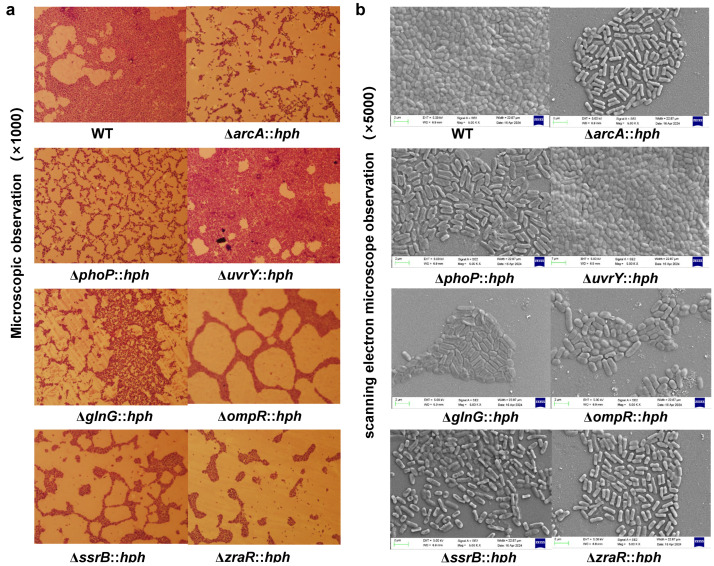
The biofilm microscopy images of *S*. Enteritidis WT and its RR mutants. (**a**) The optical microscopy images. Magnification: ×1000. (**b**) The SEM images. Magnification: ×5000, bar marker = 2 µm. The abbreviation ‘*hph*’ was employed as a screening marker for hygromycin resistance in mutants.

Optical microscopy and SEM were used to visualize the mature biofilms fixed on glass coverslip surfaces. Figure 6a,b showed the differences in the biofilm amount and architecture among the *S*. Enteritidis WT and mutants. Although both colonies of the *S*. Enteritidis WT and the RR deletion mutants contained rod-shaped cells, the WT cells were embedded in an extracellular matrix that formed basket-like networks, whereas the extracellular matrix of the six RR (ArcA, GlnG, PhoP, OmpR, ZraR and SsrB) mutants exhibited a lower adherent cell than that of WT. Of note, compared to the WT, the mutant lacking UvrY formed a solid biofilm architecture that spread evenly across the entire glass coverslip surfaces, indicating a strong biofilm formation capacity. These results suggested that the RRs ArcA, GlnG, PhoP, OmpR, ZraR, SsrB and UvrY were involved in the production of the biofilm.

### 3.6. Role of RRs in TCS in Macrocolony Biofilm Properties of S. *Enteritidis*

**Figure 7:**
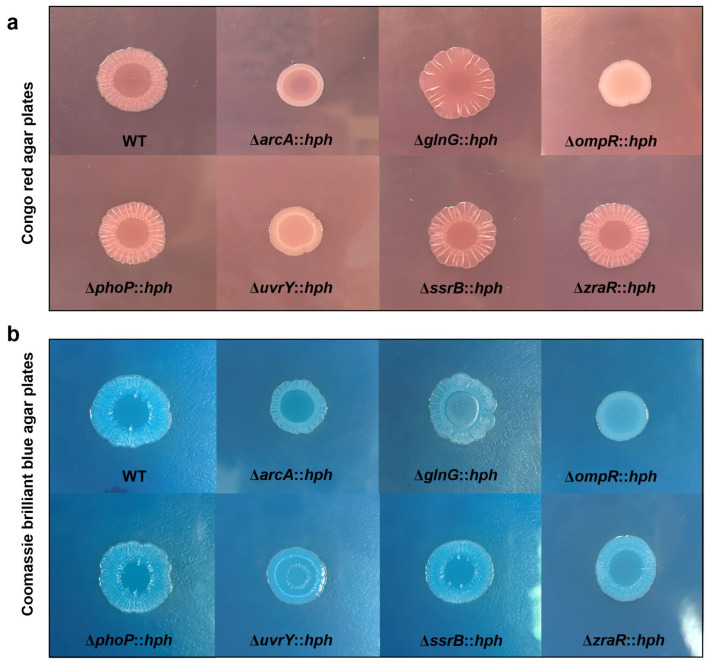
The macrocolony biofilm properties of *S*. Enteritidis WT and its RR mutants. (**a**) The different *S*. Enteritidis macrocolony phenotypes and dye-binding characteristics on CR agar plates and (**b**) CBB agar plates. The abbreviation ‘*hph*’ was employed as a screening marker for hygromycin resistance in mutants. Colony morphology images were taken after 48 h of growth at 28 °C.

Macrocolony biofilms formed on agar plates reflect the conditions of biofilms that grow on organic substrates such as soil or human food. In this study, we characterized the biofilm matrix [i.e., extracellular polymeric substances (EPS)] produced by the *S*. Enteritidis WT and RR mutants on CR and CBB plates. As shown in Figure 7a,b, the macrocolony biofilms of the different *S*. Enteritidis strains presented a similar morphology between the CR and CBB plates. Expression of curli fimbriae (a major proteinaceous component of the EPS) and cellulose nanofibers (a major polysaccharide component of the EPS) enabled a red/dry/rough morphotype of the *S*. Enteritidis WT colonies on CR agar plates (Figure 7a). However, the intensity of red color, dryness, and roughness of the surfaces varied greatly among the RR deletion mutants. The results showed that the absence of OmpR, GlnG or ArcA led to the formation of white/dry/smooth colonies, red/dry/smooth colonies and pink/dry/rough colonies, respectively, indicating a lack of curli and/or cellulose production. Moreover, the UvrY deletion mutant formed brown/smooth/sticky colonies, indicating an increased production of exopolysaccharides (the major polymer of biofilms). Although the biofilm-producing capacity of the three RR (ZraR, SsrB and PhoP) mutants was significantly decreased in comparison with the WT, no clear changes were observed in the expression of curli fimbriae and cellulose. This suggested that these RRs regulate the biofilm matrix independently of curli fimbriae or cellulose. These results emphasized the essential role of RRs of OmpR, ArcA and UvrY in regulating the production of an extracellular matrix of macrocolony biofilm.

## 4. Discussion

The high adaptability of *S*. Enteritidis led to extensive epidemic outbreaks of salmonellosis, posing a significant threat to public health [27]. The effective execution of adaptive mechanisms required the coordination of various effectors, of which TCSs were widely distributed in bacteria and played a pivotal regulatory role. Accordingly, this study sought to elucidate the role of TCS RRs in the regulation of a diverse array of stress tolerance and physiological characteristics in *S*. Enteritidis.

As food preservation often involves variations in temperature (e.g., blanching, sterilization, frozen and chilled), an increasing research interest has been focused on the genetic mechanisms that modulate bacterial temperature sensitivity/resistance. Previous studies have suggested that bacteria could encode specific TCSs to play biological roles at certain temperatures. For instance, TCS CheA-CheY was shown to be crucial for *Yersinia pseudotuberculosis* growth at 3 °C [28]; TCS CasK-CasR could regulate the expression of a desaturase gene to maintain membrane fluidity during *Bacillus cereus* growth at 12 °C [29]; *Clostridium botulinum* TCS CBO0366-CBO0365 played a role in cold tolerance via regulating metabolic pathways [30]. In this study, GlrR, the RR of TCS GlrK-GlrR, a glucosamine-6-phosphate synthesis system, was shown for the first time to play a major role in *S*. Enteritidis survival at a high temperature (50 °C), suggesting a link of glucosamine-6-phosphate synthesis to the thermo-resistance of *S*. Enteritidis. In addition, although TCS PhoQ-PhoP in *Edwardsiella* was important for bacterial virulence control, its dependent secretory protein was solely activated in a range of 23 to 35 °C and PhoQ-PhoP would be denatured at 37.9 °C [31]. Regardless of PhoQ-PhoP degeneration, the lack of PhoP had no effect on *S*. Enteritidis survival at 50 °C in this study, indicating that this TCS did not regulate *S*. Enteritidis growth under high temperatures.

Increased osmotic pressure, i.e., lowering Aw, was one of the widely used food preservation approaches. Typically, the internal osmotic pressure in bacterial cells was higher than that of the surrounding medium, which resulted in a pressure (turgor pressure) exerting outwards on the cell wall to provide a mechanical force necessary for cell elongation [32]. Therefore, bacterial cells must be able to maintain turgor despite variations in the environment. In the present study, six RR (PhoB, BaeR, CpxR, PhoP, UvrY and TctD) deletion mutants had compromised survivability compared with the *S*. Enteritidis WT under high salt conditions. Among these, BaeR and CpxR, the RRs of BaeS-BaeR and CpxA-CpxR, respectively, were known to be involved in the biogenesis, maintenance and repair of bacterial envelopes [33]; the PhoQ-PhoP system has been shown to respond to osmotic upshifts, and its RR PhoP could increase the expression of genes involved in the synthesis of cell membrane fatty acids, peptidoglycan and lipopolysaccharide to defend osmotic pressure [34,35]. Therefore, these three RRs in *S*. Enteritidis might preserve the bacterial cell membrane integrity and cell turgor under high osmotic stress, generating adaptation. Notably, for the first time, the *S*. Enteritidis RRs, including the phosphate assimilation-dependent PhoB, the carbon utilization-dependent UvrY and the tricarboxylic acids transportation-dependent TctD were found to have a role in osmotic stress adaptation, and their functions in cell membrane homeostasis require more experimental studies to elucidate.

*Salmonella* was often exposed to dramatic pH fluctuations in acid/basic foods or during intestinal colonization. To deal with these challenges, *Salmonella* has evolved complex survival strategies, of which increased accumulation of RpoS/Fur and/or activated TCSs PhoQ-PhoP and EnvZ-OmpR were defined as the major redundant systems of acid/basic tolerance [36,37]. In the present study, the absence of PhoP and OmpR had little impact on bacteria survival (deceased, statistically insignificant), but the absence of GlrR, TctD, YedW, ArcA and YehT was, for the first time, found to lead to a significant decrease in the survivability at changing pHs, indicating an important regulatory effect of these five RRs on *S.* Enteritidis adaptation to mild acid or alkalinity. In addition, ArcA, the RR of the aerobic respiration control system ArcB-ArcA, was reported to confer acid tolerance at mild acidic (pH 5.5) and murine stomach (pH 2.5) conditions in *Listeria monocytogenes* [38]. Therefore, the regulatory role of ArcA in acid tolerance might be conserved among different species.

*Salmonella* must develop an effective oxidative stress response to survive exposure to ROSs within the host cell. In this study, the presence of 5 mM H_2_O_2_ resulted in a significant decrease in the CFU of the *S*. Enteritidis WT, and this decrease was more pronounced in the six RR (PhoB, YedW, BaeR, ArcA, PhoP and UvrY) deletion mutants. The regulatory functions of PhoP in oxidative stress response have been demonstrated in our previous work, where we showed that cells lacking the PhoP regulator in *S*. Enteritidis increased the accumulation of intracellular ROSs and dysregulated redox homeostasis [35]. In addition, the functions of the above RR homologs in various bacteria on the oxidative stress response have been reported. For instance, the *Salmonella* Typhimurium (*S*. Typhimurium) and *E. coli* ArcA could regulate membrane permeability to the response ROS generated by H_2_O_2_ and HOCl, specifically by repressing the expression of multiple porin genes, including *ompD* and *ompW* [39]; the *Vibrio cholerae* (*V. cholerae*) and *E.coli* PhoB could control inorganic phosphate availability and induce the expression of catalases KatG and KatB to against ROSs [40]; the UvrY or BarA deletion mutants exhibited hydrogen peroxide hypersensitive phenotypes in *E. coli* and *Serratia marcescens FS14* [41]; the *S*. Typhimurium TCS BaeS-BaeR could regulate the expression of superoxide dismutase SodA to convert ROSs into nontoxic molecules [42]; and the *E. coli* copper and oxidation stress activator system YedV-YedW played a role in the H_2_O_2_ response’s regulation via directly controlling the expression of *hiuH* to rapidly reduced oxidative damage [43]. Thus, these RRs have a consistent function in the oxidative stress response in *S*. Enteritidis. On the other hand, the absence of QseB or PgtA significantly increased the survival rate of *S*. Enteritidis under oxidative stress. Yang et al. [44] reported that the TCS QseC-QseB mutants had significantly reduced capsule production but increased resistance to oxidative stress and osmotic pressure. In addition, the TCS PgtB-PgtA involved in the exogenous induction of phosphoglycerate transport was found to be repressed in response to the sodium hypochlorite treatment in *S*. Enteritidis [7]. Therefore, QseB and PgtA played a negative role in oxidative stress adaptation in *S*. Enteritidis, and the mechanism underlying their regulation is a focus of our future work.

*Salmonella* moves through liquid by swimming motility and across a surface by swarming motility, which enables cells to reach a favorable environment. Such motility is known to be related to flagellar assembly/rotation and pathogenicity. In the present study, we found that the absence of ArcA, GlnG or PgtA abolished the motility of *S*. Enteritidis with non-motile phenotypes in swim agar. It is well-known that TCS GlnL-GlnG (also known as NtrB-NtrC) plays an important role in maintaining bacterial nitrogen utilization and biological nitrogen fixation, while the regulatory function of ArcB-ArcA is one of the mechanisms that enabled bacteria to adapt to changing oxygen availability [45,46]. Recently, *Acidovorax citrulli* NtrC and *Plesiomonas shigelloides* ArcA were reported to activate the expression of the pili-related gene *pilA* and the flagella- and chemotaxis-related genes *flaK*, *rpoN* and *cheV*, respectively [45,47]. Therefore, the positive regulatory function of the RRs ArcA and GlnG in swimming motility was conserved across species. Additionally, although our findings emphasized the importance of TCS PgtB-PgtA for the motility of *S*. Enteritidis, the functions of this TCS on bacteria phenotypes were still sparse. On the other hand, five RRs (PhoB, UhpA, OmpR, UvrY and QseB) were identified as having a negative regulatory role in *S*. Enteritidis’ swarming motility; however, the regulatory details of these TCSs differed among species and conditions. It was reported that TCS UhpB-UhpA, one of the glucose 6-phosphate transport systems, could negatively regulate *Edwardsiella piscicida’s* motility and its colonization in the intestine of tilapia [48]; TCS EnvZ-OmpR repressed flagellar master operon *flhDC* expression, and thus limited *E. coli* motility, which was in contrast to the positive role it played in *Yersinia enterocolitica* [48,49]. Kostakioti et al. [50] reported that phosphorylated QseB (QseB-P) in enterohemorrhagic *E. coli* activated the expression of *flhDC*, while QseB-P in uropathogenic *E. coli* inhibited *flhDC* expression, and the *P. aeruginosa* and *V. cholerae* PhoB could upregulate the Rhl quorum sensing system or operon *acgAB* expression, promoting hyper swarming [51,52]; TCS BarA-UvrY was critical for carbon utilization, and the swarming motility of uropathogenic *E. coli* was diminished in its *uvrY* mutant via reducing *flhCD* expression [53]. As a result, this study highlighted the importance of these RRs in motility and their strain-dependent contributions, as well as their regulatory role in the flagellar master operon *flhDC*.

Motility was closely related to biofilm formation positively for a long time because the ability of biofilm formation was a surface-associated behavior that begins with the initial attachment of bacterial cells to a surface, and this process involves flagella-mediated motility. In this study, the absence of ArcA or GlnG led to a decrease in both biofilm formation capacity and motility, whereas the absence of UvrY led to an increase in these two characteristics of *S*. Enteritidis, suggesting that RRs ArcA, GlnG and UvrY mediated a positive correlation between *S*. Enteritidis biofilm formation and the motility in a flagella-dependent way. However, the absence of PhoP, OmpR, ZraR or SsrB led to a decrease in biofilm-forming ability on both polystyrene and glass coverslip surfaces but no significant changes in motility. Therefore, other factors could influence the development of biofilms as well. In addition, the biofilm was a complex microbial community encased in extracellular polymeric substances. In this study, microscope observation and CR/CBB agar plate analysis demonstrated that those RRs could also affect the biofilm structure and composition of cellulose and curli, an extracellular macromolecule that composed biofilm, thus promoting biofilm formation. Among these, OmpR was known for its contribution to the transition from the reversible to the irreversible attachment phase in biofilm development by increasing the synthesis of type I fimbriae and curli in *E. coli* [54]; SsrB was a master biofilm regulator that activates *csgD* (*agfD*) in *S*. Typhi and *S*. Typhimurium [55]; PhoP played a role in controlling the expression of c-di-GMP related genes (*csgD* and *adrA*) to develop complete biofilms in the presence of bile in *S*. Typhimurium [56]; in *V. cholerae*, ArcA could directly activate the expression of *vpsT*, which was a positive regulator for biofilm matrix production by directly sensing cyclic di-GMP, whereas NtrC negatively regulated biofilm formation by inhibiting the expression of *vpsT* [57,58]. Therefore, the effect of RRs on bacterial biofilm formation was also closely linked to their regulatory mode of the expression of c-di-GMP-related genes. It was notable that ZraR, the RR of the ZarS-ZarR system, was involved in metal tolerance-resistant processes, but the significance of its role in biofilm formation was reported for the first time. Taken together, these observations suggested that RRs have critical regulatory functions in controlling the motility and/or extracellular matrix contents and can promote or repress biofilm formation in *S*. Enteritidis.

## 5. Conclusions

This study characterized the functions of RRs in TCS in the formation of stress tolerance, motility and biofilm. The results found that there were one, five, six and eight RRs in the TCS involved in the response and adaptation of high temperature, pH stress, high osmolarity and oxidation conditions, respectively. Additionally, eight and seven RRs in TCS, respectively, were found to contribute to the motility and biofilm formation of *S*. Enteritidis. Of note, certain RRs (ArcA, PhoB and UvrY) regulated multiple stress adaptation and/or physical characteristics. These TCSs constituted a complex network of regulations that facilitated adaptation to a large variety of stress factors from the environment, which may be intrinsic to *S*. Enteritidis. This study lays a foundation for the development of therapeutic targets and preventive measures to control *S*. Enteritidis during the food process. Nevertheless, further investigation is needed into the function of these RRs in other species and food environments. Additionally, determining the molecular mechanisms of these RRs represents an exciting avenue for future research.

## Data Availability

The original contributions presented in the study are included in the article/Supplementary Materials, further inquiries can be directed to the corresponding author.

## Supplementary Materials

The following supporting information can be downloaded at: https://www.mdpi.com/article/10.3390/foods13223709/s1, Figure S1: The relative viability of *S.* Enteritidis strains (relative to WT) that survived at 37 °C.
