# Supplementary material for: Roles of Response Regulators in the Two-Component System in the Formation of Stress Tolerance, Motility and Biofilm in Salmonella Enteritidis
Source: Foods. 2024 Nov 20;13(22):3709. doi: 10.3390/foods13223709 (PMC11594007; doi:10.3390/foods13223709)
Supplement: Supplementary file 1 [file foods-13-03709-s001.zip › foods-3305172-supplementary.pdf]

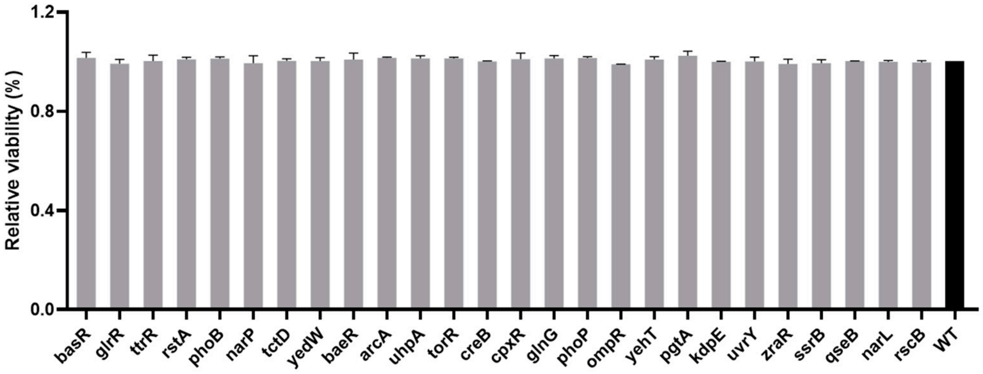

Figure S1. The relative viability of *S*. Enteritidis strains (relative to WT) that survived at 37°C. The various RR mutants were labelled according to the RR-encoding gene on the X-axis. Data was presented as mean ± standard deviation.
